# Preliminary Trajectories in Dietary Behaviors during the COVID-19 Pandemic: A Public Health Call to Action to Face Obesity

**DOI:** 10.3390/ijerph17197073

**Published:** 2020-09-27

**Authors:** Roberta Zupo, Fabio Castellana, Rodolfo Sardone, Annamaria Sila, Vito Angelo Giagulli, Vincenzo Triggiani, Raffaele Ivan Cincione, Gianluigi Giannelli, Giovanni De Pergola

**Affiliations:** 1 Population Health Unit “Salus in Apulia Study”—National Institute of Gastroenterology “Saverio de Bellis”, Research Hospital, 70013 Bari, Italy; castellanafabio@hotmail.it (F.C.); rodolfo.sardone@irccsdebellis.it (R.S.); annamaria.sila@irccsdebellis.it (A.S.); gdepergola@libero.it (G.D.P.); 2 Interdisciplinary Department of Medicine—Section of Internal Medicine, Geriatrics, Endocrinology and Rare Diseases, School of Medicine, University of Bari “Aldo Moro”, 70121 Bari, Italy; vitogiagulli58@gmail.com; 3 Section of Internal Medicine, Geriatrics, Endocrinology and Rare Disease, Interdisciplinary Department of Medicine, School of Medicine, University of Bari, 70121 Bari, Italy; vincenzo.triggiani@uniba.it; 4 Department of Clinical and Experimental Medicine, University of Foggia, 71122 Foggia, Italy; ivan.cincione@unifg.it; 5 Scientific Direction, National Institute of Gastroenterology “Saverio de Bellis”, Research Hospital, 70013 Bari, Italy; gianluigi.giannelli@irccsdebellis.it; 6 Clinical Nutrition Unit, Department of Biomedical Science and Human Oncology, University of Bari, School of Medicine, Policlinico, 70121 Bari, Italy

**Keywords:** COVID-19, diet, dietary changes, body weight, obesity

## Abstract

The world is currently struggling to face the coronavirus pandemic (COVID-19), and many countries have imposed lockdowns and recommended quarantine to limit both the spread of the virus and overwhelming demands for medical care. Direct implications include the disruption of work routines, boredom, depression, increased calorie consumption, and other similar harmful effects. The present narrative review article briefly analyzes the preliminary effects of the quarantine lifestyle from the standpoint of dietary habits. In six different databases, we searched for original articles up to 10 August 2020, assessing eating habits among populations during the COVID-19 pandemic, and recorded any change in the intake of major food categories, as well as changes in body weight. The research strategy yielded 364 articles, from which we selected 12 articles that fitted our goal. Our preliminary findings revealed a sharp rise of carbohydrates sources consumption, especially those with a high glycemic index (i.e., homemade pizza, bread, cake, and pastries), as well as more frequent snacks. A high consumption of fruits and vegetables, and protein sources, particularly pulses, was also recorded, although there was no clear peak of increase in the latter. Data concerning the consumption of junk foods lacked consistency, while there was a decreased alcohol intake and fresh fish/seafood consumption. As a possible connection, people gained body weight. Therefore, in the realistic perspective of a continuing global health emergency situation, timely preventive measures are needed to counteract obesity-related behaviors in the long-term, so as to prevent further health complications.

## 1. Introduction

Globally, as of 19 July 2020, there were 14,043,176 confirmed cases of Coronavirus Disease (COVID-19) reported to the World Health Organization (WHO), including 597,583 deaths [1]. Many countries imposed a stringent lockdown in order to enforce social distancing and prevent the spread of infection [2]. The use of social distancing, face masks, gloves, and other individual protection measures is having a massive impact on reducing the current peak of active cases but, as recently demonstrated, over time, a decreasing sense of alarm about the pandemic may contribute to a new, larger second wave of epidemics [3]. In any case, the enforced confinement has upset our life priorities and changed the way we live in several ways, ranging from working behaviors (e.g., smart working, job cuts) to the psychological field, fostering the onset of depression, boredom, sedentary activities, and several more harmful effects on life habits [4,5]. Moreover, constantly hearing or reading about the pandemic during quarantine without a break has stressfully affected most people. Stress and boredom are both drivers of overeating, as people resort to sugary “comfort foods,” resulting in the introduction of more energy/calories and an increased craving for food [6]. The latter is a kind of feeling-state driven by emotional (intense desire to eat), behavioral (seeking food), cognitive (thoughts about food), and physiological (salivation) sensations [7]. In this context, the introduction of carbohydrate-rich foods in particular may be an unconscious form of self-medication against adverse stimuli, stimulating serotonin release, which positively affects the mood. Interestingly, the effect of carbohydrate craving in increasingly low moods is proportional to the food glycemic index. The closely involved downside is that these unhealthy dietary behaviors promote the development of obesity, inducing a chronic systemic inflammatory status that, concurrently with other chronic non-communicable conditions such as dyslipidemia, hypertension, heart diseases, diabetes, and lung disease, may increase the risk of more severe complications due to COVID-19 [8]. In this regard, a recent Italian snapshot showed that patients with excess weight admitted to a medical ward for COVID-19-related pneumonia, even younger ones, more frequently needed assisted ventilation and access to intensive or semi-intensive care units than normal weight patients [9]. Sleep disorders also contribute to worsen the quarantine-related scenario, further worsening stress and increasing food intake, thus giving rise to a dangerous vicious cycle [10].

From the perspective of the current stress-overeating situation, nutritional approaches have become a primary goal to counteract the onset of risky dietary behaviors. In this light, we conducted this narrative synthesis with the aim of identifying a preliminary trend of dietary changes during the period of lockdown, also highlighting some findings concerning weight changes, so as to stimulate a call to action to combat the obesity issue within the pandemic. This preliminary synthesis, however, also hopes to stimulate research aimed at defining the risk of COVID-19 infection and the consequent mortality of people affected by obesity, as well as providing food for thought to better orientate risk management, both remotely (e.g., by investing in training healthcare professionals dedicated to nutritional counseling) and in common clinical practice.

## 2. Methods

**Figure 1:**
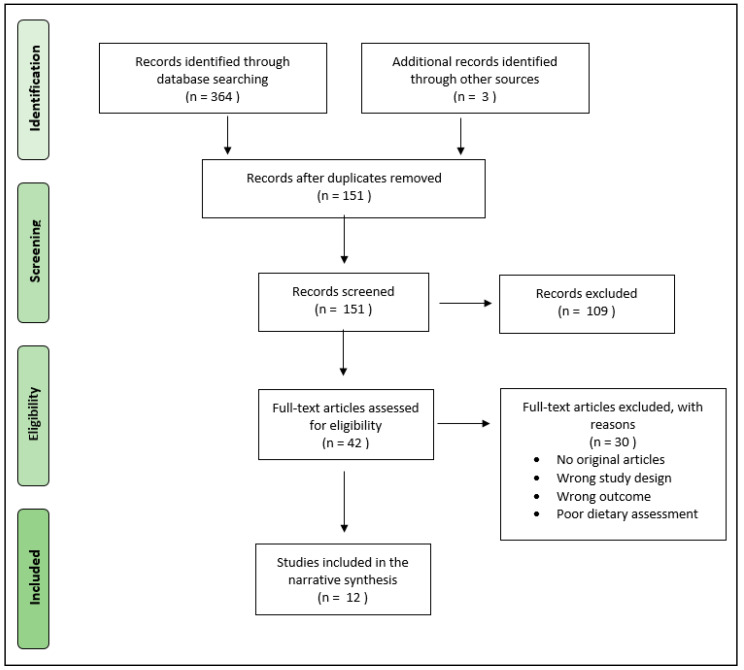
Preferred Reporting Items for Systematic reviews and Meta-Analyses (PRISMA) flow diagram of search procedure.

The present was a narrative review article. We performed separate searches in the US National Library of Medicine (PubMed, Bethesda, MD 20894, USA), Medical Literature Analysis and Retrieval System Online (MEDLINE), EMBASE, Scopus, Ovid, and Google Scholar databases to find original articles on dietary changes and behaviors during the COVID-19 pandemic. The search strategy used in PubMed and MEDLINE and adapted to the other four electronic sources is shown in Table 1. A PRISMA flow diagram of the search procedure is also provided (see Figure 1). The references of the included studies were analyzed for additional references of interest. The last search for the purposes of the present narrative review was performed on 10 August 2020. No language restriction was adopted. Given the novelty of the topic, and therefore small number of data, no skimming was applied to the study design, and both longitudinal and cross-sectional observations were included. For the same reason, no age range was applied to the selected study populations. Two investigators (R.Z., F.C.) independently, and in duplicate, searched for papers, screened titles and abstracts of the retrieved articles, reviewed the full-texts, and selected articles for inclusion. The following inclusion criteria were applied: original studies assessing dietary habits among populations during the COVID-19 pandemic with no discrimination between methods of administration (online questionnaire or phone interview) or for estimating intake (daily or weekly frequency). Technical reports, letters to the editor, and systematic and narrative review articles were excluded.

The following information was extracted independently and in duplicate by the same investigators in a piloted form: (1) general information in the selected studies (study design, sample size, country, methods for assessing diet, and estimating intake); (2) items derived from the nutritional survey (changes in body weight, carbohydrate intake, junk foods, dressing fats, protein sources, snacks, as well as alcohol intake, and also information about home-cooking practice). The data on carbohydrate consumption were divided by key categories into four subgroups according to major categories (sugary foods, fruit, vegetables, and cereals). Likewise, we sorted the “weight changes” item into “gain,” “loss,” and “none” based on whether any difference in body weight occurred during the lockdown period. Consistency in the reported items for the assessment of dietary habits among selected studies was verified. Data were cross-checked, any discrepancy was discussed, and disagreements were resolved by a senior researcher (RS).

## 3. Results

### 3.1. Overview of Selected Studies

**Figure 2:**
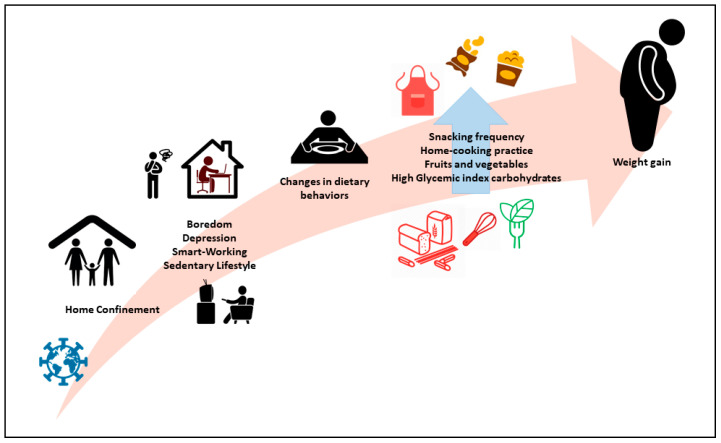
Food trajectories triggered by the COVID-19 home confinement.

In total, 12 studies were identified for the purpose of this narrative review. Table 2 summarizes descriptive characteristics of the included studies. The longitudinal setting characterizing a third (four out of 12) of the selected studies allows them to be accurately compared before and during the COVID-19 dietary settings. Meanwhile, the cross-sectional approach of the remaining studies records offers a snapshot of the dietary settings and may possibly strengthen the longitudinal observations. Daily or weekly frequency of foods consumption was adopted to assess changes in foods intake, using phone interviews or online questionnaires. Carbohydrate sources (sugary foods, fruit, vegetables and cereals), fast foods, dressing fats, protein sources (pulses, meat, dairy products, fish), snacks, and alcoholic beverages consumption were evaluated as major food items. Some surveys showed a slight lack of completeness, due to the heterogeneous nature of the investigations. In addition, we observed that half of the adopted study surveys reported on the practice of cooking meals at home. Seven studies reported about changes in body weight, as “gain,” “loss,” or “no change.” Table 3 and Table 4 summarize the dietary characteristics of the study findings, subdivided by principal foods intake, and including body weight changes, home-cooking practice, and overall findings. Figure 2 represents food trajectories triggered by the COVID-19 home confinement. Lastly, some information about reported changes in physical activity level is provided in Table 5.

### 3.2. Single Foods or Food Groups

Both longitudinal and cross-sectional observations revealed an overall increased consumption of carbohydrates sources. Starting from sugary foods, such as sugary drinks, ice creams, and homemade or packaged sweet foods (i.e., cakes, biscuits, chocolate), we observed a rise in daily/weekly consumption by up to 70%. The same increase resulted for fruit and vegetables consumption, except for the Polish population reported both by Sidor and Rzymski [11] and by Górnicka et al. [12]. In the first case, nearly one-third of the 1097 Polish citizens surveyed did not consume fresh vegetables and fruits on a daily basis, while the same proportion admitted to consuming sweets at least once every day. In the second, about 20% of the 2381 reported a decreased daily consumption. Among studies investigating cereals intake (rice, grains, flours, and derived products such as pizza or bread), an increase in daily/weekly consumption was evident. In this regard, the only longitudinal observation, carried out by Ghosh et al. on a fairly small Indian sample, revealed a 21% increase in the daily consumption of grains and rice [13]. Similarly, seven cross-sectional observations showed that a large majority consumed higher amounts of cereals [11,12,13,14,15,16,17]. In particular, homemade pizza, fresh bread, pasta, and rice were found to rule the trend among the Italian and Spanish surveys performed over a wide range of ages and sample sizes (increases by up to 40% and 39%, respectively) [14,15]. The large sample of Poles surveyed by Górnicka et al. [12] likewise reported an increase in whole grain products consumption by more than 16%.

Changes in fat consumption were mainly assessed by recording the intake of dressing fats (olive oil, ghee, butter). It should be noted that although none of the cross-sectional studies reported this information, longitudinal observations showed a slight over-consumption, particularly referred to Indian [13] and Spanish populations [18]. In both these cases, the increased daily consumption as compared to the pre- COVID-19 era did not exceed 5%.

Eleven studies reported major protein sources (fish, meat, eggs, pulses, and dairy products). Some of them reported a low-moderate increase in pulses consumption. Among longitudinal observations, an increased pulses consumption was declared by about 23.6% of Indians surveyed (>4 times per week) versus 22.8% before COVID-19, and 7% of Spaniards (more times/week than before COVID-19). Similarly, cross-sectional observations showed a higher consumption by Polish [11,12] and Italian populations [14]. Particularly, Górnicka et al. reported that low-fat meat, eggs, pulses, and dairy products consumption increased up to 20%, while there was a decrease of processed meat consumption (17.7%) [12]. In line, Di Renzo reported an overall increase up to 15% of these four protein sources without discriminating between types of meat [14].

By contrast, studies inquiring about fresh fish and seafood consumption reported an overall decrease in weekly frequency intake (by 22% and 33% among Italians and Spaniards respectively, and by 17% among Poles). Information about meat consumption lacked consistency, despite an apparent trend towards a slight increase. In this regard, one of the Italian surveys reported an apparently increased daily red meat consumption (from 1.80 ± 1.53 to 3.46 ± 2.45 servings/day) in 41 children and adolescents from Verona within a longitudinal setting [19]. A similar cross-sectional observation on a larger sample size, including subjects aged more than 12 years old belonging to every region, reported an increased egg, meat, and pulse consumption by up to 15% [14]. Similarly, the majority (81%) of the overweight Italians surveyed by Pellegrino et al. [16] showed equal or greater consumption of overall protein sources than pre-COVID-19. Again, a quarter of the 700 Chilean investigated by Reyes-Olavarría et al. declared that they consumed red and white meat more than 3 times/week [20]. Conversely, the longitudinal Spanish observation registered a decrease of meat daily intake by almost 2% [18].

As to eggs and dairy products, although this was not sufficiently investigated, the confinement induced a slightly increased consumption among Indian [13], Italian [14], Polish [11,12], and Spanish [15] populations.

### 3.3. Junk Foods, Snacks, and Alcoholic Beverages

There is still no clear trend for junk food intake during the current pandemic. Among longitudinal observations, Ruiz-Roso et al. found an 18.5% increase in the weekly frequency of junk food consumption, surveying 820 adolescents from Spain, Italy, Brazil, Colombia, and Chile [21]; the same positive direction regarding potato chips intake among Italian adolescents was found by Pietrobelli et al. [19]. In contrast, other studies reported a reversed trend, probably due to the age group investigated. In fact, data from adult Spanish and Italian populations showed an overall decline in the consumption of this food category (34% and 29.8% less, respectively).

Particularly noteworthy is that confinement encouraged a significant increase in snack consumption apart from the main daily meals, and this finding was demonstrated in all studies published thus far, regardless of the age group surveyed. The number of people who consumed snacks more than 4 times a day increased by 23% in India [13], while 37.6% of Spaniards [18] and 30% of Chinese [17] increased their overall snacking frequency. Instead, the snapshot of Italians and Poles recorded increases in the frequency of consumption by about 60% [16] and 23% [22], respectively.

All studies examining the changes in alcoholic beverages consumption concluded that this dropped, except for a Polish survey (showing a 14.6% and 18.1% increase). In detail, 8% of the Indians and 13% [14] and 36.8% [22] of the Italians reduced their daily intake by 25–50%. Similarly, the two Spanish surveys reported a 57.3% decrease in the weekly frequency of consumption in the longitudinal observation by Rodríguez-Pérez et al. [18], and a 27% decrease in the cross-sectional evaluation carried out by Romeo-Arroyo et al. [15].

### 3.4. Body Weight Changes, Physical Activity Level, and Home-Cooking Habits

Five of the 12 studies did not report any information about changes in body weight within the populations surveyed. However, the available evidence allows us to assume that almost 50% of people maintained their starting weight, without undergoing any changes. A considerable proportion gained weight: 19% of Indians [13], 12% of Spaniards [18], 29.9% of Poles [11], 32% of Chileans [20], and 48.6% [14] and 19.5% [22] of Italians. The few data available on people who lost weight reported a 33% loss among Indians [13], 32% among Chileans [20], 18.6% among Poles [11], and 3.9% among Italians [14,22].

As predicted due to the enforced confinement, the level of physical activity recorded a drastic drop to 79% (Table 5), and this finding likely contributed to the weight trajectories.

Home-cooking became much more common, being practiced by 97% of Indians [13] and the majority of the Spanish population. An ample increase was also registered in Poland (62.3% and 48%) [11,12] and Chile (59.6%) [20].

## 4. Discussion

In this narrative synthesis analyzing the latest data on people’s food choices during the COVID-19 confinement periods, the main finding was an initial decline in overall dietary lifestyle. Specifically, the consumption of carbohydrates sources, as well as snacking and home-cooking habits, underwent a substantial increase. Instead, a lack of consistency of data concerning the consumption of meat and junk foods was noted, probably due to the paucity of studies and the heterogeneous age range of the populations surveyed, as well as assessment methods. Moreover, these preliminary data showed a slightly increased consumption of pulses, fruit, and vegetables, and a great increase in the habit of cooking meals at home. As a possible connection, people gained some weight, even if almost half of those surveyed did not declare significant fluctuations. The finding that, as a result of the enforced confinement, people had more desire to cook, and above all to knead, is of particular concern. Thus, the consumption of homemade desserts, bread and pizza, as well as sweet pastries (biscuits, cakes, and other bakery) rapidly rose, inducing a higher exposure to unhealthy foods with a high glycemic index [23]. In other words, these unhealthy dietary habits facilitate a poor control of the body weight, leading to obesity. This is undoubtedly a spontaneous consequence of stress and/or boredom and/or other kinds of negative emotions (so-called “emotional eating”), as opposed to a more limited level of physical activity closely related to the confinement. Moreover, people tended to eat snacks more frequently, and foods outside meals in general, even if they were not really hungry, just as a way to kill boredom. Another key point is the inability to procure fresh food, as people were forced to stay at home, order takeout food, and prefer long shelf-life foods so as to minimize going out to procure groceries. In fact, the limited access to daily grocery shopping led to a reduced consumption of fresh foods in favor of highly processed ones, such as convenience foods, junk foods, snacks, and ready-to-eat cereals, which tend to be high in fats, sugars, and salt. This inclination was matched by the overall drop in fresh fish and seafood intake. The latter, besides being a noble source of protein that is useful in controlling body weight [24], is a well-known source of omega-3 fatty acids, specifically eicosapentaenoic acid (EPA) and docosahexaenoic acid (DHA), with anti-inflammatory effects that could enhance resistance and the recovery from Severe Acute Respiratory Syndrome CoronaVirus-2 (SARS-CoV-2) [25]. Similarly, fresh vegetables and fruits consumption was no less affected, as reported by the Polish survey. This issue may be extremely worrying considering the great emphasis allocated by the latest findings to vitamins and minerals deficiencies, including beta-carotene, vitamin C, and vitamin E, especially in view of their antioxidant and anti-inflammatory properties. In fact, the inadequate intake of these micronutrients is associated with both obesity and impaired immune responses, thus making people more susceptible to viral infections [26,27].

Preliminary data on meat consumption lack consistency, despite an apparent trend towards a slight increase, while eggs and pulses intake seemed to be mildly increased, perhaps because of their easier availability and longer shelf-life compared to fish or meat. The trajectories for junk foods consumption, apparently increased among younger people, are equally controversial. This practice is likely promoted by the possibility of home delivery of food during this period. Lastly, alcoholic beverages consumption registered a general decrease, possibly due to the lack of “social drinking” (i.e., enjoying drinks with friends at bars) or just of spending time with other people. Noteworthily, since a heavy alcohol intake has been demonstrated to weaken the immune system and thus reduce the ability to cope with infectious diseases [28], this may be positive from the long-term emergency perspective.

### Strengths and Limitations

It is important to highlight the strengths and limitations of the present review. First of all, the limited number of studies weakens the weight of the findings. Secondly, for the same reason, we included two different types of settings, without any skimming. Thirdly, the studies featured different and mostly poorly accurate dietary assessment approaches; indeed, quantification in grams was uncommon. However, this is the first review paper to describe preliminary dietary modifications during the COVID-19 pandemic, and can thus offer early food for thought on this matter.

## 5. Conclusions

Summing up these preliminary data, it is not surprising that a fair percentage of people experienced an increase in body weight. This may pose a severe problem in the highly realistic perspective of a continuing global health emergency situation. Nutrition has become a priority right now, because a well-balanced diet is the best way to get all the essential nutrients we need to ensure normal immune function, while reducing the risk of obesity. More research is urgently needed to better understand the global dietary trend during the COVID-19 emergency in the long term. However, according to these initial data, preventive measures must already be put in place in order to implement basic tools against obesogenic lifestyles within the COVID-19 era. Actions to address the growing challenge of obesity require systematic and multi-sector policy actions, as well as recognition that governments need to address the roots of obesity. A starting point would be to implement comprehensive policies and actions to improve the food environment, for example through restrictions on marketing and taxes. Concurrently, it would be advisable to support research defining the risk of contracting the COVID-19 infection and subsequent mortality in people living with obesity, in addition to providing clear guidance on managing risk factors. Additionally, it would be desirable to provide specific training for health professionals to enable them to support people living with obesity in the COVID-19 era, so as to minimize related risks and unconscious prejudices, as well as to prevent stigma.

## Figures and Tables

**Table 1:** Search strategy used in the US National Library of Medicine (PubMed) and Medical Literature Analysis and Retrieval System Online (MEDLINE), according to the selected descriptors.

Strategy	Descriptors Used
# 1	(diet[tiab]) OR (feeding[tiab])) OR (habits[tiab]) OR (dietary lifestyle[tiab]) OR (dietary habits[tiab]) OR (dietary [tiab]) OR (dietary pattern[tiab]) OR (dietary behavior[tiab]) OR (food[tiab]) OR (foods[tiab]) OR (food habits[tiab]) OR (nutritional habits[tiab]) OR (eating habits[tiab]) OR (eating[tiab])
# 2	(change[tiab]) OR (changes[tiab]) OR (modifications[tiab]) OR (alterations[tiab]) OR (alteration[tiab]) OR (different[tiab]) OR (differences [tiab])
# 3	(sars cov 2[tiab]) OR (covid 19[tiab]) OR (severe acute respiratory syndrome coronavirus 2[tiab])
# 4	# 1 AND # 2 AND # 3

**Table 2:** Descriptive characteristics of included studies.

Study Details							
Ref.	Authors, Year	Sample Size	Study Group	Study Design	Country	Method of Estimating Intake	Dietary Assessment Method
[11]	Sidor et al., 2020	1097	18–71 years	Cross-sectional	Poland	Daily/weekly frequency	Online questionnaire
[12]	Górnicka et al., 2020	2.381	18+ years	Cross-sectional	Poland	Daily/weekly frequency	Online questionnaire
[13]	Ghosh et al., 2020	150	Middle-aged adults	Longitudinal	India	Daily frequency	Phone interview
[14]	Di Renzo et al., 2020	3533	12–86 years	Cross-sectional	Italy	Daily/weekly frequency	Online questionnaire Eating Habits and Lifestyle Changes in COVID19 lockdown (EHLC-COVID-19)
[15]	Romeo-Arroyo et al., 2020	600	18–68 years	Cross-sectional	Spain	Weekly frequency	Online questionnaire
[16]	Pellegrini et al., 2020	150	18–75 years	Cross-sectional	Italy	Daily/weekly frequency	Online questionnaire
[17]	Wang et al., 2020	2289	18–81 years	Cross-sectional	China	Weekly frequency	Online questionnaire
[18]	Rodríguez-Pérez et al., 2020	7514	>18 years	Longitudinal	Spain	Daily/weekly frequency	Online questionnaire
[19]	Pietrobelli et al., 2020	41	6–18 years	Longitudinal	Italy	Daily frequency	Phone interview
[20]	Reyes-Olavarría et al., 2020	700	18–62 years	Cross-sectional	Chile	Daily/weekly frequency	Online questionnaire
[21]	Ruiz-Roso et al., 2020	820	10–19 years	Longitudinal	Italy, Spain, Chile, Colombia, Brazil	Weekly frequency	Online questionnaire (National School Health Survey—PeNSE survey)
[22]	Scarmozzino et al., 2020	1.929	−	Cross-sectional	Italy	Weekly frequency	Online questionnaire

**Table 3:** Dietary characteristics of the study findings by principal carbohydrate sources (sugary foods, fruit, vegetables, and cereals), body weight changes (gain, loss, none) and overall findings.

Authors, Year [Ref.]	Body Weight Changes			Carbohydrate Sources				Overall Findings
	Gain	Loss	None	Sugary Food	Fruit	Vegetables	Cereals	
Ghosh et al., 2020 [13]	19%	33%	48%	7% reported 25–50% more sugar intake	20% reported 25–50% more fruits intake	9% increased servings/day (3 or more)	21% increased cereals intake (rice, grains)	Increased carbohydrate, snacking and fruit intake, and home cooked meals
Ruiz-Roso et al., 2020 [21]	-			47.4% increased sweet foods intake (>4/week) versus 40.6% pre-COVID-19. Ditto for sugar beverages (23.8% against 22.7%)	58.6% increase (>4/week) versus 53.9% pre-COVID-19	70.8% increase (>4/week) versus 66.2% pre-COVID-19	-	Increased pulses, fruit, and vegetables intake, and home cooked meals. Higher sweet food intake. The overall diet quality did not improve.
Rodríguez-Pérez et al., 2020 [18]	12%	-	47%	Up to 21% decreased daily sweet beverages intake	Up to 18% increased daily intake	Up to 19% increased daily intake	-	Higher intake of fruits, vegetables, and pulses and lower intake of red meat, alcohol, and fried foods.
Pietrobelli et al., 2020 [19]	-			Increase of sugary drinks (0.40 ± 0.90 to 0.90 ± 1.16 servings/day)	Increased (1.16 ± 0.74 to 1.39 ± 0.70 servings/day)	Increased (1.34 ± 0.74 to 1.27 ± 0.69 servings/day)	-	No changes in reported vegetables intake. Fruit intake increased. Potato chip, red meat, and sugary drink intakes increased significantly.
Sidor et al., 2020 [11]	29.9%	18.6%	-	One-third consumed at least once or more/day	One-third did not consume fresh vegetables and fruits on a daily basis	One-third did not consume fresh vegetables and fruits on a daily basis	The majority (64.2%) consumed grains once or more/day	One-third of people surveyed did not consume fresh vegetables and fruits on a daily basis, while the same proportion admitted to consuming sweets at least once every day. Obese people surveyed tended to eat vegetables, fruits, and pulses less frequently, and salty foods, meat, and dairy more often.
Di Renzo et al., 2020 [14]	48.6%	13.9%	37.4%	43% increased homemade sweets	37.4% increase	37.4% increase	Up to 40% increased (homemade pizza, fresh bread, cereals)	Increased homemade foods (e.g., sweets, pizza and bread), cereals, and pulses, and decreased fresh fish, packaged sweets and baked products, delivery foods and alcohol intake
Romeo-Arroyo et al., 2020 [15]	-			Over 50% increased sweets intake	35% increase	30% increase	Increase of cereals (20%), pasta/rice (39%), and bread (36%) consumption	Increased baking, fruits, and vegetables intake
Scarmozzino et al., 2020 [22]	19.5%	-	50.7%	42.5% increased chocolate, cakes, and ice creams	21.2% increase	21.2% increase	-	Increased consumption of fresh fruit and vegetables. Decreased alcohol consumption
Górnicka et al., 2020 [12]	-			39.9% increased homemade pastries intake, 8.4% decreased sugar-sweetened beverages, and 5% decreased energy drink intake	20.1% decreased servings/day consumption	Almost 19% decreased servings/day consumption	16.3% increased whole grain products intake	Highly increased consumption of homemade pastries. Increased consumption of eggs, pulses, and cereals, as well as alcohol. Decreased fish, fruit, and vegetable intake.
Reyes-Olavarría et al., 2020 [20]	32%	17%	51%	-	30.9% increased daily intake	30.9% Increased daily intake	-	Increased fruit and vegetables consumption, and home cooked meals. Higher junk and fried foods intake. The overall diet quality did not improve.
Pellegrini et al., 2020 [16]	Self-reported weight and BMI significantly increased by 1.51 kg			72% reported equal or greater sweets consumption than pre-COVID-19	81% reported equal or greater consumption than pre-COVID-19	81% reported equal or greater consumption of cereals than pre-COVID-19	Increased frequency of overall food intake. More sweets and snacks consumption.	
Wang et al., 2020 [17]	-				30% reported consuming more vegetables and fruit, especially women	Increased consumption (250–400 g/day), especially men	Higher intake of fruits, vegetables, and cereals. Increased snacking frequency.	

**Table 4:** Dietary characteristics of the study findings by other major principal food sources. Data on home-cooking habits were also included.

Authors, Year [Ref.]	Junk/Fast Foods	Dressing Fat	Protein Sources	Snacks	Alcohol	Home Cooking
Ghosh et al., 2020 [13]	-	5% increased overall fat intake (ghee, butter)	3% increased overall protein intake (eggs, fish, meat, pulses, soybean)	23% increase snacking frequency (>4/day)	8% decreased daily intake by 25–50% versus 3% of increase	Widespread (97%)
Ruiz-Roso et al., 2020 [21]	Up to 18.5% increase (>4/week)	-	23.6% increased pulses intake (>4/week) against 22.8% before	-		
Rodríguez-Pérez et al., 2020 [18]	Up to 34% lower fast foods and processed meat weekly intake	4% increase of olive oil daily intake	Up to 75% and 7% increase in weekly fish and pulses intake. Almost 2% decreased daily meat intake.	37.6% increased snacking frequency	57.3% decreased weekly intake.	45.7% increase
Pietrobelli et al., 2020 [19]	Increased potato chips intake (0.07 ± 0.24 to 0.61 ± 0.83 serving/day)	-	Increased daily red meat consumption (from 1.80 ± 1.53 to 3.46 ± 2.45)	-		
Sidor et al., 2020 [11]	Higher frequency	-	Dairy, meat, and pulses were consumed by the majority quite often during the week	Up to 60% increase	14.6% increase	62.3% increase
Di Renzo et al., 2020 [14]	29.8% decrease	-	Up to 15% increased eggs, meat, and pulses. Decreased fresh fish consumption -22%	-	13% decreased wine and beer consumption	-
Romeo-Arroyo et al., 2020 [15]	-		30% increase of eggs, 39% increase of milk and dairy products, and 33% decrease of fish intake	-	27% decreased alcoholic beverages	Widespread (between 4 and 6 on a 7-point scale of agreement)
Scarmozzino et al., 2020 [22]	-			23.5% increased frequency	36.8% decreased wine, beer, and liquors consumption	-
Górnicka et al., 2020 [12]	36.6% decrease	-	Increased low-fat meat and/or eggs (15.7%), pulses (13.9%), and milk and dairy products (20.8%) intake. Decreased fish and seafood (17%), and processed meat (17.7%) intake.	19.7% decreased salty snacks	18.1% increase	48% increase
Reyes-Olavarría et al., 2020 [20]	Increased junk and fried foods intake (1–2 times/week)	-	About 25% consumed red and white meat more than 3 times/week. 75.1% and 83.7% consumed fish and pulses 1–2 times/week.	-	30% declared a daily consumption of alcohol	59.6% increase
Pellegrini et al., 2020 [16]	-		81% reported equal or greater consumption of overall protein sources than pre-COVID-19	60.6% reported equal or greater snacking frequency	-	
Wang et al., 2020 [17]	-		Meats, dairy products, and eggs fulfilled the recommendation of the dietary guidelines for Chinese residents	About 30% reported an increased snacking frequency	-	

**Table 5:** Details about reported trends on changes in physical activity level during the COVID-19 lockdown.

Ref.	Authors, Year	Physical Activity Level	Study Design
[11]	Sidor et al., 2020	-	Cross-sectional
[12]	Górnicka et al., 2020	43% decreased	Cross-sectional
[13]	Ghosh et al., 2020	42% decreased	Longitudinal
[14]	Di Renzo et al., 2020	No significant difference between the percentage of people that did not train before (37.7%) or during (37.4%) the COVID-19 lockdown. However, a higher frequency of training during the emergency was found when compared to the previous period.	Cross-sectional
[15]	Romeo-Arroyo et al., 2020	-	Cross-sectional
[16]	Pellegrini et al., 2020	79.3% did not do any physical activity or reduced their physical activity level compared to pre-COVID-19	Cross-sectional
[17]	Wang et al., 2020	More than 50% decreased	Cross-sectional
[18]	Rodríguez-Pérez et al., 2020	59.6% decreased	Longitudinal
[19]	Pietrobelli et al., 2020	Sports time decreased significantly by 2.30 ± 4.60 h/week	Longitudinal
[20]	Reyes-Olavarría et al., 2020	The highest percentage of subjects passed ≥6 h sitting or sedentary activities (54.4%)	Cross-sectional
[21]	Ruiz-Roso et al., 2020	-	Longitudinal
[22]	Scarmozzino et al., 2020	-	Cross-sectional

## Abbreviations

COVID-19	Coronavirus Disease 19
WHO	World Health Organization
MEDLINE	Medical Literature Analysis and Retrieval System Online
EPA	Eicosapentaenoic Acid
DHA	Docosahexaenoic Acid
PRISMA	Preferred Reporting Items for Systematic Reviews and Meta-Analyses
